# Efficacy and safety of transurethral split of prostate for benign prostatic hyperplasia: a meta-analysis

**DOI:** 10.1186/s12894-020-00704-4

**Published:** 2020-09-03

**Authors:** Yiyu Huang, Jiaxin LI, Shan Yang, Daozhang Yuan, Shusheng Wang

**Affiliations:** 1 Department of Urology, GuangDong Second Traditional Chinese Medicine Hospital, No.212, Luogang Road Street, Luogang District, Guangzhou, 510700 Guangdong China; 2 Department of Colorectal Surgery, The Sixth Affiliated Hospital, Sun Yat-Sen University, Guangzhou, 510655 China; 3 Department of Emergency, Guangdong Provincial Hospital of Chinese Medicine, Guangzhou, 510120 China; 4 Department of Urology, Guangdong Provincial Hospital of Chinese Medicine, No.261, Datong Road, Yuexiu District, Guangzhou, 510030 Guangdong China

**Keywords:** Transurethral split of prostate, TUSP, Benign prostatic hyperplasia, BPH, Meta-analysis

## Abstract

**Background:**

Transurethral resection of the prostate (TURP) is the first choice for the treatment of benign prostatic hyperplasia. However, Transurethral split of prostate (TUSP) also seems to have clear clinical efficacy and clinical promotion value. To better clarify the potential and limitations of this treatment of prostate hyperplasia. This study objectively evaluated the clinical efficacy and safety of TUSP.

**Methods:**

The Pubmed, Cochrane Library, Embase, China National Knowledge Infrastructure (CNKI), Database for Chinese Technical Periodicals (VIP), Wanfang (Wanfang data), and SinoMed databases were searched for relevant studies. We then used Revman Manager 5.3 to perform a meta-analysis of all randomized controlled trials that evaluated the efficacy and safety of TUSP versus the classic surgical procedures commonly used in the clinic.

**Results:**

A total of 7 studies involving 592 patients were included. The combined data showed that TUSP can shorten the operation time [MD: -33.68; 95% CI: − 38.45 to − 28.91; *P* < 0.001], reduce intraoperative blood loss [MD: -56.06; 95% CI: − 62.68 to − 49.43; *P* < 0.001], shorten the time of indwelling catheter [MD: -1.83; 95% CI: − 1.99 to − 1.67; *P* < 0.001], shorten the postoperative hospital stay length [MD: -1.61; 95% CI: − 1.90 to − 1.32; *P* < 0.001] and improved postoperative quality of life score (QOL) [MD: 0.16; 95% CI: 0.02 to 0.29; *P* = 0.02] compared to traditional surgical approaches. There were no statistically significant differences in international prostate symptom score (IPSS), maximum urinary flow rate (Qmax), residual urine volume (RUV), or complications between TUSP and traditional approached.

**Conclusion:**

TUSP can be an effective alternative for clinical treatment of benign prostatic hyperplasia. Given the limitations of the included studies, more high-quality randomized controlled trials are needed in the future to validate or update the results of this analysis.

## Background

Benign prostatic hyperplasia (BPH) is one of the most common benign diseases in middle-aged men. The incidence of the disease is positively correlated with age. According to statistics, the histological prevalence rates is approximately 10% for men aged 30–40, 20% for men aged 41–50, 50 to 60% for men aged 60–70, and 80 to 90% for men aged 70–90. Lower urinary tract symptoms (LUTS), such as frequent urination, urgency, and dysuria, are the main pathological features of BPH [1]. Extremely high morbidity and the resultant LUTS have brought much pain to the lives of male patients. If the treatment is not appropriate or timely, it may also be complicated with upper urinary tract damage or even affect dual renal function [2]. Currently, transurethral resection of the prostate (TURP) is the clinically preferred treatment [3, 4]. However, there are still complications such as hemorrhage, transurethral resection syndrome (TURS) and retrograde ejaculation [5]. New surgical methods, such as bipolar transurethral electrovaporization of the prostate (TUPKP) and transurethral plasmakinetic enucleation of the prostate (TUKEP), have emerged and have been shown to have relatively few complications. However, these methods also have the problem of expensive equipment and high technical requirements for doctors, which is not conducive to clinical promotion [6]. Therefore, it is necessary to find an effective, relatively safe and inexpensive surgical method. Professor Guo Yinglu from the First Hospital of Peking University, China, has been working on the transurethral split of prostate (TUSP) method for many years and finally developed a variety of models of double-chambered water balloon expansion catheters. They standardized the surgical procedure, making the surgery more mature in the treatment of BPH, and proved that the operation is safe and effective through related research [7, 8].

Although some Chinese scholars have conducted randomized controlled trials (RCTs) on using TUSP to treat BPH and found that it has relatively clear clinical efficacy and clinical promotion value, small sample size made it is impossible to provide a comprehensive evaluation. To date, there has been no systematic review and meta-analysis of the efficacy and safety of TUSP in the treatment of BPH. Therefore, we systematically searched and analyzed existing literature to assess the efficacy and potential advantages of TUSP.

## Methods

This meta-analysis were following the Cochrane Handbook of Systematic Reviews of Interventions and was conducted in accordance with the Preferred Reporting Items for Systematic Reviews and Meta-Analyses guidelines [9, 10].

### Search strategy

To obtain the relevant research, we comprehensively searched the Pubmed, Cochrane Library, Embase, China National Knowledge Infrastructure (CNKI), Database for Chinese Technical Periodicals (VIP), Wanfang (Wanfang data), and SinoMed database from inception until April 2019. The combined search used the Medical Subject Heading (MeSH) terms and non-MeSH terms, such as “prostatic hyperplasia”, “BPH”, “transurethral prostate division”, “TUSP”, and “randomized controlled trial”. All articles were viewed and read independently by two authors (YYH and JXL), and any objections were submitted to another investigator (SSW) who was not involved in the initial process. References were also manually searched for additional studies in the relevant original and commentary articles.

The inclusion criteria were as follows: (1) randomized control design; (2) full-text content and related data can be obtained; (3) the study provides accurate data that can be analyzed, including the total number of subjects and the valuable results of each indicator. In addition, the exclusion criteria were as follows: (1) duplicate studies, case reports, conference abstracts, animal experiments, editorial opinions; (2) insufficient data for research, such as a lack of means and standard deviations; (3) case-control study or cohort study; and (4) lack of parallel control.

### Study selection and data extraction

Two investigators independently screened the literature based on the inclusion and exclusion criteria, and if there were differences, discussed the resolution or resolved it by consulting a third party. The extracted data included the first author’s name, research characteristics (publication year, duration, and design), participant characteristics (mean age and sample size), baseline status, interventions, outcome measures, and adverse events.

### Assessment of risk of Bias in included studies

The RCT bias risk assessment tool of the Cochrane Collaboration was used to evaluate the quality of the included studies. Specific items included selection bias (random sequence generation, allocation concealment), implementation bias (blind method of implementers and participants), measurement Bias (blindness in outcome assessment), incomplete outcome data, publication bias (selective reporting), and other biases. The reviewers evaluated each item as “low risk of bias”, “high risk of bias” or “unclear of bias” based on the specific details of the study. Disagreements in quality evaluation were resolved through discussions between researchers.

### Statistical analysis

Meta-analysis of the data was performed using RevMan 5.3 (Cochrane Collaboration, Oxford, UK) [11] provided by the Cochrane Collaboration. The relative risk (risk ratio, RR) and the 95% confidence interval (95% CI) were used to calculate the effect size of the dichotomous data. The effect sizes for continuous variable were presented as the mean difference (MD) and 95% CI. Heterogeneity test was assessed by the chi-square test, and the test efficiency was set to α = 0.05. When the heterogeneity test result was not statistically significant (*P* > 0.05, *I*^*2*^ < 50%), the fixed effect model (FEM) was used. When the heterogeneity test results were statistically significant (*P* < 0.05, *I*^*2*^ > 50%), a random effects model (REM) was used. Sensitivity analysis was used if necessary to find the sources of heterogeneity, and subgroup analyses were used to reduce heterogeneity.

## Results

### Description of studies

**Fig. 1:**
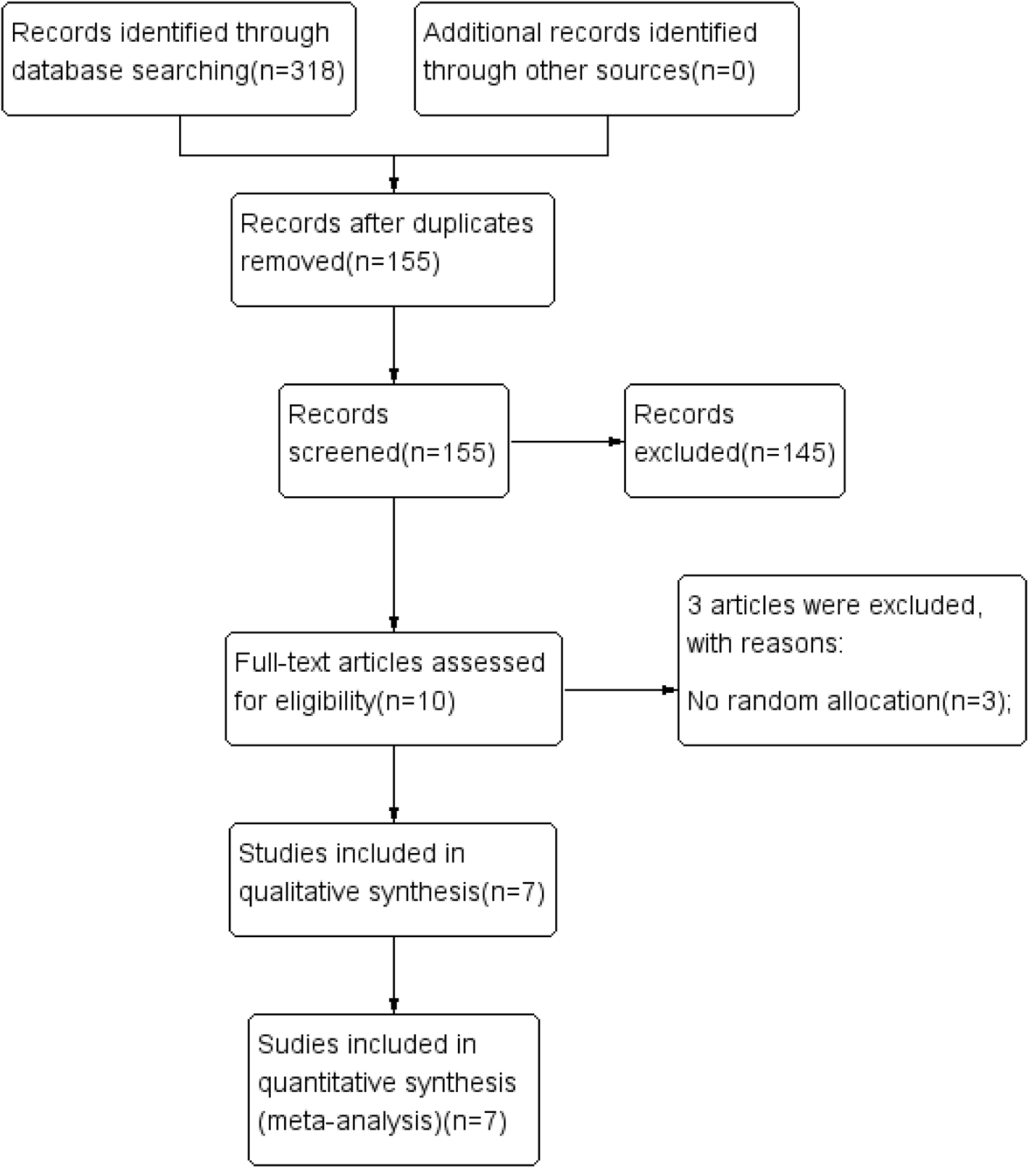
Flow diagram of literature searches according to the Preferred Reporting Items for Systematic Reviews and Meta-Analyses statement

Figure 1 shows the flow chart for the search process and study selection. Through a comprehensive search, a total of 318 articles were obtained. After excluding 163 duplicate articles, 155 articles were included. After screening the titles and abstract, 145 articles were excluded because they did not meet the inclusion criteria for the following reasons: retrospective analysis (60), non-clinical studies (3), and irrelevant studies (76). Finally, we carefully read the full text of the remaining 10 articles, and 3 articles were excluded because they did not use random assignment.

### Study characteristics and quality of evidence

A total of 7 studies involving 592 patients were included in the meta-analysis [12–18]. Table 1 describes the basic characteristics of the included studies. These studies were published between 2017 and 2019. Four of the studies compared TUSP with TUPKP [13, 14, 16, 17], two of studies compared TUSP with TUKEP [12, 18], and another study compared TUSP with TURP [15]. The outcome measures involved in this meta-analysis included operation time, intraoperative blood loss, postoperative indwelling catheter time, postoperative hospital stay length, IPSS score at 3 months postoperatively, QOL at 3 months postoperatively, Qmax at 3 months postoperatively, RUV at 3 months postoperatively and complications. All of these tests were conducted in China.

**Table 1:** Characteristics of the included studies

Author	Year	Sample size (EG/CG)	Age(Y)		Intervention methods		Follow-times	outcomes
			EG	CG	EG	CG		
Kong Min [12]	2017	40/40	NA	NA	TUSP	TUKEP	3 months	Operation time (min); Intraoperative blood loss (ml); IPSS; Qmax;
Wang Qi [13]	2017	50/50	74.4 ± 9.8	73.1 ± 9.4	TUSP	TUPKP	3 months	Operation time (min); Intraoperative blood loss (ml); Postoperative indwelling catheter time(d); Postoperative hospital stay length(d); IPSS; Qmax; QOL; RUV; Complications
Zhou Jin [14]	2018	30/30	79.21 ± 15.37	78.86 ± 15.25	TUSP	TUPKP	NA	Operation time (min); Intraoperative blood loss (ml); Complications
Kong Qingkuo [15]	2018	107/103	NA	NA	TUSP	TURP	3 months	Operation time (min); Postoperative indwelling catheter time(d); Postoperative hospital stay length(d); IPSS; Qmax; QOL; RUV; Complications
Li Hong [16]	2018	15/15	67.4 ± 10.1	69.1 ± 10.5	TUSP	TUPKP	3 months	Operation time (min); Intraoperative blood loss (ml); Postoperative indwelling catheter time(d); Postoperative hospital stay length(d); IPSS; Qmax; QOL; RUV; Complications
Liu Shuzhi [17]	2018	10/10	72.58 ± 12.42	74.85 ± 12.28	TUSP	TUPKP	12 months	Operation time (min); Intraoperative blood loss (ml); Postoperative indwelling catheter time(d); Postoperative hospital stay length(d);
Wang Bo [18]	2019	46/46	69.3 ± 3.8	69.8 ± 3.7	TUSP	TUKEP	3 months	Postoperative indwelling catheter time(d); Postoperative hospital stay length(d);IPSS; Qmax; Complications

*Qmax* maximum urinary flow rate, *RUV* residual urine volume, *IPSS* international prostate symptom score, *QOL* quality of life

**Fig. 2:**
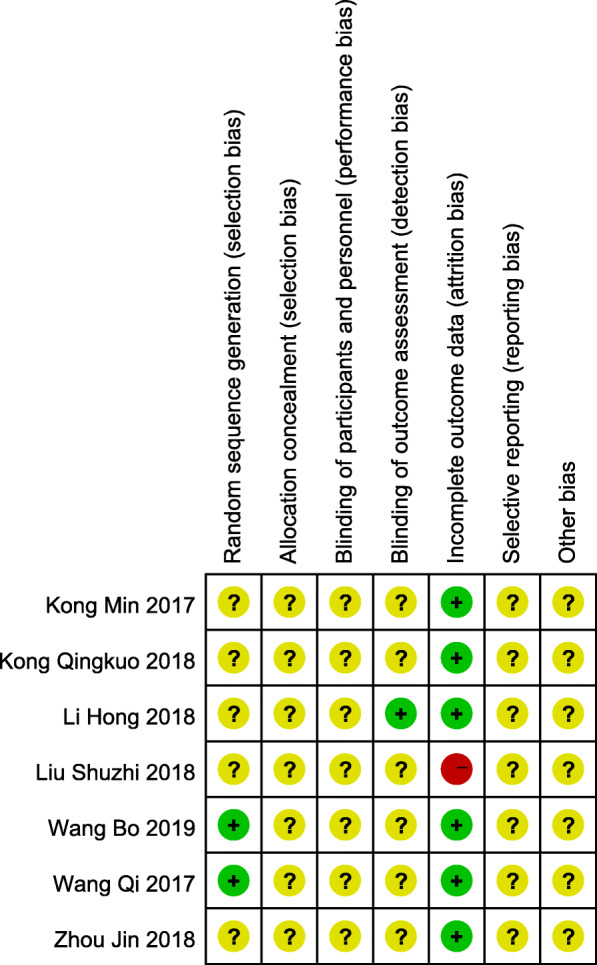
Risk of bias summary of included studies

**Fig. 3:**
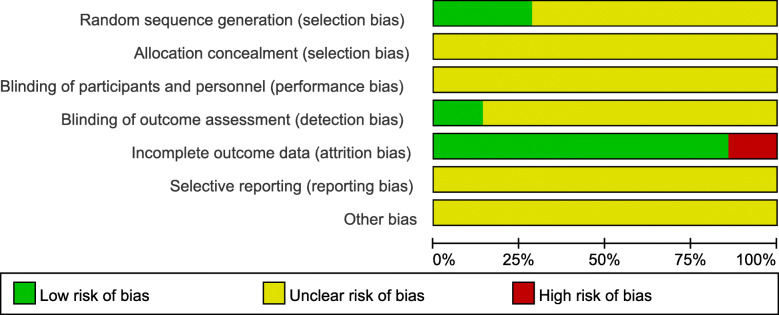
Evaluation for bias risk of included studies

For the included 7 studies, the bias was largely unclear. Figure 2 shows a summary of the methodological quality of each of the included studies. The judgment of the reviewers for each item of risk of bias tool is shown in Fig. 3 as a percentage.

### Operation time

**Fig. 4:**
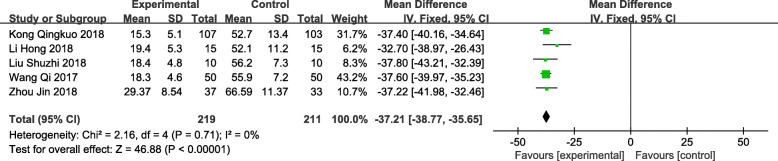
Forest plot and meta-analysis of Operation time (min). Experimental: the group of TUSP; Control: the group of traditional clinical surgery

A total of 6 trials [12–17] reported on the operation time, involving a total of 510 patients (259 in the observation group and 251 in the control group). The results showed that TUSP required a shorter time for surgery than traditional clinical surgery [MD:-33.68; 95% CI: − 38.45 to − 28.91; *P* < 0.001], but there was a large amount of heterogeneity (*P* < 0.001, *I*^*2*^ = 88%). We performed a sensitivity analysis, which revealed that the source of the heterogeneity may have been trials conducted by Kong Min [12]. By reading the full text, we found that the study group underwent endoscopic observation of the surgical field after TUSP treatment, leading to prolonged operation time in the observation group, which may have resulted in heterogeneity. After eliminating these trials, we found no heterogeneity between the groups (*P* = 0.71, *I*^*2*^ = 0%), so a fixed effect model was adopted for the meta-analysis. The time required for TUSP in the treatment of benign prostatic hyperplasia was shorter, and the difference was statistically significant [MD: -37.21; 95% CI: − 38.77 to − 35.65; *P* < 0.001) (Fig. 4).

### Intraoperative blood loss

**Fig. 5:**
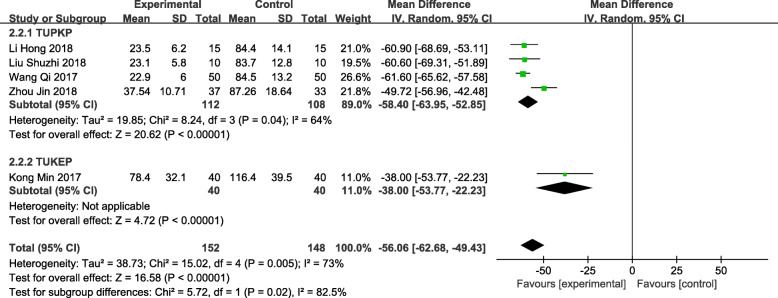
Forest plot and meta-analysis of Intraoperative blood loss (ml). Experimental: the group of TUSP

A total of 5 trials [12–14, 16, 17] described intraoperative blood loss, involving a total of 300 patients (152 in the observation group and 148 in the control group). The heterogeneity test indicated that the data had high level of heterogeneity (*P* = 0.005, *I*^*2*^ = 73%), but the sensitivity analysis did not find any sources of heterogeneity, and the meta-analysis results were more stable. Therefore, the random effects model was used for analysis. The results showed that the amount of bleeding in the treatment of benign prostatic hyperplasia was lower in the TUSP group, and the difference was statistically significant [MD: -56.06; 95% CI: − 62.68 to − 49.43; *P* < 0.001]. Subgroup analysis based on the different surgical methods of the control group showed that TUSP can reduce intraoperative blood loss compared with TUPKP or TUKEP (Fig. 5).

### Postoperative indwelling catheter time

**Fig. 6:**
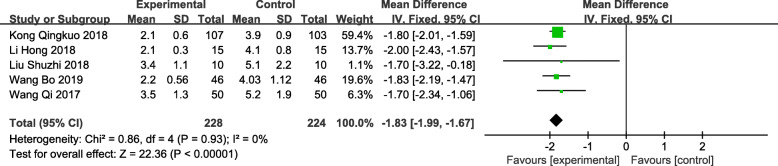
Forest plot and meta-analysis of Postoperative indwelling catheter time(d). Experimental: the group of TUSP; Control: the group of traditional clinical surgery

A total of 5 trials [13, 15–18] recorded postoperative urinary catheter indwelling time, including 228 patients in the observation group and 224 patients in the control group. The meta-analysis showed that compared with traditional clinical surgery, the time of indwelling catheter after TUSP was shorter, and the difference was statistically significant [MD: -1.83; 95% CI: − 1.99 to − 1.67; *P* < 0.001) (Fig. 6). There was no heterogeneity among the studies (*P* = 0.93, *I*^*2*^ = 0%).

### Postoperative hospital stay length

**Fig. 7:**
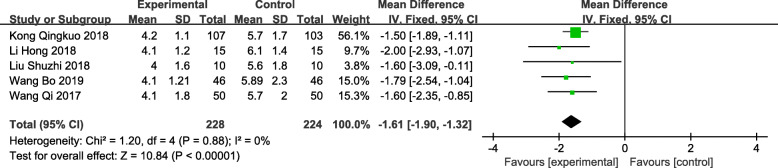
Forest plot and meta-analysis of Postoperative hospital stay length(d). Experimental: the group of TUSP; Control: the group of traditional clinical surgery

A total of 5 trials [13,15–18] reported on postoperative hospital stay length, involving a total of 452 patients (228 in the observation group and 224 in the control group). The final results showed that compared with traditional clinical surgical treatment, patients with benign prostatic hyperplasia after TUSP treatment had a significantly shorter postoperative hospital stay length [MD: -1.61; 95% CI: − 1.90 to − 1.32; *P* < 0.001] (Fig. 7). There was no heterogeneity between the studies (*P* = 0.88, *I*^*2*^ = 0%).

### IPSS score at 3 months postoperatively

**Fig. 8:**
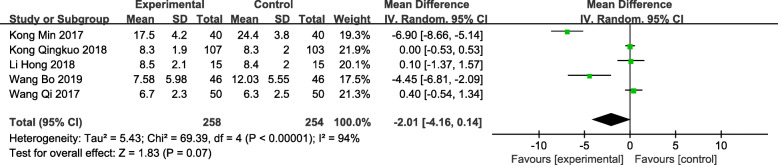
Forest plot and meta-analysis of IPSS scores at 3 months postoperatively. Experimental: the group of TUSP; Control: the group of traditional clinical surgery

Five of the included trials [12, 13, 15, 16, 18] recorded the patient’s IPSS score at 3 months postoperatively, involving a total of 512 patients. The results showed that there was no significant difference in the IPSS score between TUSP and traditional clinical surgery at 3 months postoperatively [MD: -2.01; 95% CI: − 4.16 to − 0.14; *P* = 0.07) (Fig. 8).

### Qmax at 3 months postoperatively

**Fig. 9:**
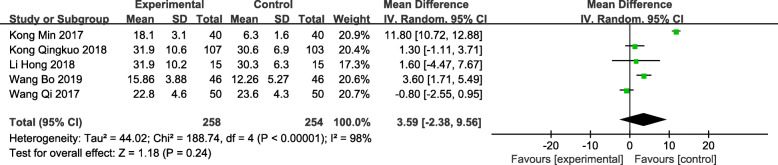
Forest plot and meta-analysis of Qmax at 3 months postoperatively. Experimental: the group of TUSP; Control: the group of traditional clinical surgery

A total of 5 trials [12, 13, 15, 16, 18] compared the patient’s Qmax at 3 months postoperatively, with 258 patients in the observation group and 254 patients in the control group. There was no significant difference in the Qmax between TUSP and traditional clinical surgery at 3 months postoperatively [MD: 3.59; 95% CI: − 2.38 to 9.56; *P* = 0.24] (Fig. 9).

### QOL scores at 3 months postoperatively

**Fig. 10:**
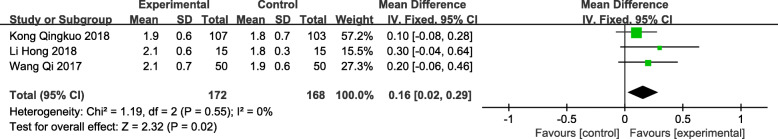
Forest plot and meta-analysis of QOL scores at 3 months postoperatively. Experimental: the group of TUSP; Control: the group of traditional clinical surgery

There were 3 trials [13, 15, 16] that examined the QOL scores at 3 months postoperatively, involving 340 patients (172 in the observation group and 168 in the control group). The meta-analysis showed that the QOL scores of patients after TUSP were higher, and the difference was statistically significant [MD: 0.16; 95% CI: 0.02 to 0.29; *P* = 0.02] (Fig. 10). There was no heterogeneity between the studies (*P* = 0.55, *I*^*2*^ = 0%).

### RUV at 3 months postoperatively

**Fig. 11:**
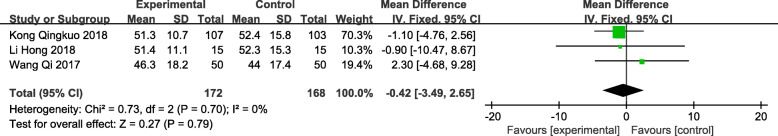
Forest plot and meta-analysis of RUV at 3 months postoperatively. Experimental: the group of TUSP; Control: the group of traditional clinical surgery

There were 3 trials [13, 15, 16] that examined RUV at 3 months postoperatively, involving 340 patients (172 in the observation group and 168 in the control group). The meta-analysis results showed no significant difference in RUV between TUSP and traditional clinical surgery [MD: -0.42; 95% CI: − 3.49 to 2.65; *P* = 0.79) (Fig. 11).

### Complications

**Fig. 12:**
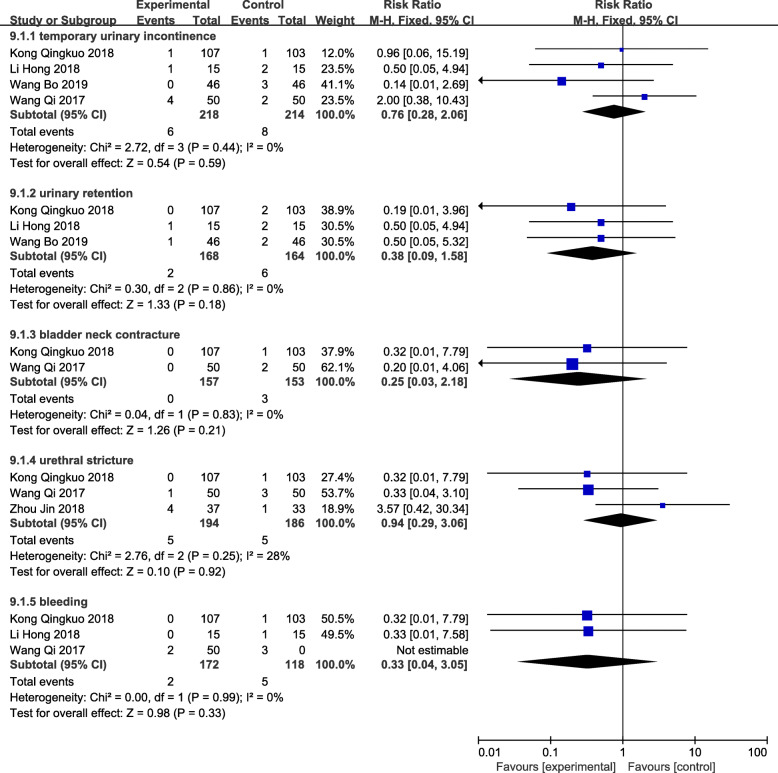
Forest plot and meta-analysis of Complications. Experimental: the group of TUSP; Control: the group of traditional clinical surgery

Complications were recorded in 6 trials [12–16, 18]. Among them, Kong Min’s study only recorded the total incidence of complications, including 1 case of complications in the observation group and 7 cases in the control group. The incidence of complications in the treatment group was significantly lower than that of the control group. Figure 12 shows an analysis of the complications in the other five trials. The results showed that there were no statistical differences between the two types of temporary urinary incontinence, urinary retention, bladder neck contracture, urethral stricture and secondary bleeding.

### Publication Bias analysis

We used the Egger test of Stata 15.1 to assess the possibility of publication bias. Egger’s test of QOL indicated that there was significant publication bias (*P* = 0.011) and the other outcome indicators have no significant publication bias.

## Discussion

Benign prostatic hyperplasia is one of the most common diseases in middle-aged men. The clinical manifestations of prostate enlargement and lower urinary tract symptoms are extremely troublesome for the patient’s life and work. As the global population ages, the incidence of this disease is increasing [19, 20]. At present, it is difficult to achieve satisfactory results using simple drug treatments; surgery is the most effective way to treat BPH, and traditional transurethral resection of the prostate is considered the gold standard for the treatment of BPH [21]. However, traditional clinical surgery is highly invasive, and it has shortcomings such as long operation time, large amounts of intraoperative blood loss, TURS and high treatment costs [22], so it is difficult to be widely used in developing countries. Therefore, we urgently need a safer and cheaper surgical method to treat BPH.

In 1984, with the development of interventional radiology, Burhenne et al. used arteriographic balloon catheters to perform prostate balloon dilatation on 10 male cadavers, which proved that the procedure can increase the diameter of the prostatic urethra. Additionally, the author used this method to perform TUDP to himself, verifying the potential value of TUDP [23]. In 1987, Castaneda performed prostate balloon dilatation on 12 BPH patients and achieved satisfactory results. The application of this surgical procedure was reported for the first time [24]. Because of the simplicity and convenience of this operation, the complications are relatively few. This has been positively received by most clinicians, prompting a large number of doctors to conduct more research on this technology [25]. Unfortunately, Gill [26] and McLoughlin [27] did not achieve satisfactory results in clinical studies of TUDP. Lepor [28] compared TUDP and cystoscopy and found that they have the same effect on symptom responses. Further clinical studies have shown that, since the therapeutic effect of TUDP on BPH is uncertain, even with a low incidence of complications, TURP is still the best choice for BPH [29]. This has led doctors and patients to gradually lose confidence in TUDP.

However, because this surgical method is relatively inexpensive and suitable for use in developing countries such as China, Huang [7, 8] has improved TUDP and redesigned the balloon catheter. The new operation can crack the prostate capsule. Therefore, it was named transurethral split of the prostate (TUSP), and the efficacy and safety of TUSP were verified in animal experiments and clinical trials.

The main principle of TUSP is to expand the prostatic capsule at the 12 o’clock position of the apex of the prostate through the internal balloon to reduce the urethral closure pressure. Then, the external balloon continues to expand the entire dorsal capsule. Finally, a wide “U”-shaped urethra extending to the 12 o’clock position is formed, which reduces the tension of the urethral wall [30]. To obtain a better curative effect, there are certain requirements. First, the appropriate catheter type should be selected strictly according to the prostate volume before operation. If necessary, surgeons should observe the prostate shape and size with and endoscope first to accurately determine the type. Second, the catheter should be stabilized to prevent slippage during the operation. The inner balloon is fixed to the membranous part, and the external balloon is fixed to the prostatic urethra [7]. Some Chinese scholars use ultrasound-guided TUSP surgery, which greatly improves the accuracy of positioning and the success rate of surgery [31]. Finally, it is advisable to stabilize the pressure of the internal and external balloon of the catheter at 0.3 MPa.

TUSP simply dilates the obstructed urethral prostate on the basis of preserving the prostate organs, and it has less trauma and simple operation. It is especially suitable for patients who require the preservation of sexual function, are accompanied by a variety of underlying diseases, are poorly tolerated by anesthesia, or are elderly. However, there are still some limitations. First, TUSP can cause mechanical laceration of the prostate urethra, which may cause bleeding during the operation. The catheter has no hemostatic function, so electrocoagulation hemostasis may be needed during the operation. Secondly, TUSP is unavailable for viewable operation [8], which may require further research and development of visual and automatic instruments. Finally, it is impossible to collect prostate tissue for histological examination because TUSP operation is performed while preserving prostate function [8].

After 328 patients were followed up for 38–99 months, Huang [8] found only 2 cases of recurrent dysuria, which showed that TUSP had a good long-term curative effect. However, due to the limited amount of literature, it is expected that more research on long-term linkages will be published in the future.

The successful application of TUSP in the treatment of patients with BPH may be associated with a significant reduction in inflammatory infiltration and collagen content in the bladder neck, prostatic urethra and urethra. Bianchi-Frias [32] and Huang [7] found in experiments on mice and dogs that the inflammatory infiltration and collagen content in the ageing prostate is very rich. When the dog is subjected to TUSP, inflammatory infiltration and collagen in the bladder neck, prostatic urethra and urethra are significantly reduced. These factors may weaken the contraction of the prostate tissue and expand the urethra. However, the mechanism remains to be confirmed by further research.

The purpose of this review is to analyze the efficacy and safety of TUSP in the treatment of benign prostatic hyperplasia. We searched the existing literature and included 7 randomized controlled trials of TUSP in the treatment of BPH. The results of the analysis showed that compared with the traditional surgical approach, TUSP can shorten the operation time, reduce the amount of intraoperative blood loss, shorten the time of indwelling catheter and speed up the postoperative recovery. At the same time, we also compared IPSS, Qmax, RUV, QOL and complications at 3 months after the operation. The results showed that except for the higher QOL score after TURP, the IPSS, Qmax, RUV and complications of the two treatments were not statistically significant. Therefore, from a clinical point of view, TUSP leads to less trauma, can shorten the operation time, and has relatively high safety. It is suitable for patients who are intolerant or unwilling to undergo surgical resection of the prostate, such as elderly or physically weak patients.

Although this review included a systematically search and analysis, there are still some limitations. First, the sample size was small, and not all outcomes are reported in each of the included studies, which may affect the accuracy of the results. Second, due to the insufficient number of articles included in the literature, this study classified TURP, TUPKP and TUKEP into the traditional clinical surgery approach as a control group, which may lead to the emergence of heterogeneity. We hope that more research can be carried out in the future. Third, only one study had a follow-up period of 12 months, while other studies had a follow-up period of 3 months. Therefore, the long-term effectiveness and safety of TUSP could not be evaluated. It is strongly recommended that future studies use longer follow-up periods. Fourth, due to the particularity of the surgery, the surgical approach requires the consent of the patient and the family, so the included studies were not double-blind. Finally, the included studies were all from China, so the evaluation of TUSP in this review may only be applicable to Chinese people. We look forward to future clinical research in more countries.

## Conclusions

Compared with traditional prostatectomy, TUSP may have a similar curative effect on BPH, and it has the advantages of reduced trauma, less intraoperative blood loss, shorter operation times and shorter hospital stays. In the Chinese population, it is an effective and safe surgical procedure for patients who are old or weak and unable to tolerate prostatectomy. It is worth promoting. However, given the small number of studies included in this meta-analysis and the low quality, this conclusion requires further validation from more high-quality clinical randomized controlled trials.

## Data Availability

The datasets used and/or analyzed during the current study are available from the corresponding author on request.

## Abbreviations

BPH: Benign prostatic hyperplasia
TUSP: Transurethral split of prostate
TURP: Transurethral resection of the prostate
TUPKP: Bipolar transurethral electrovaporization of the prostate
TUKEP: Transurethral plasmakinetic enucleation of the prostate
TURS: Transurethral resection syndrome
QOL: Quality of life score
IPSS: International prostate symptom score
Qmax: Maximum urinary flow rate
RUV: Residual urine volume
MD: Mean difference
RR: Risk ratio
CI: Confidence interval
RCTs: Randomized controlled trials
